# Role of Nutritional Factors at the Early Life Stages in the Pathogenesis and Clinical Course of Type 1 Diabetes

**DOI:** 10.1155/2015/382165

**Published:** 2015-03-26

**Authors:** Yukiko Kagohashi, Hiroki Otani

**Affiliations:** ^1^Department of Health and Nutrition, The University of Shimane, Matsue, Shimane 690-0044, Japan; ^2^Department of Developmental Biology, Faculty of Medicine, Shimane University, Izumo, Shimane 693-8501, Japan

## Abstract

Nutrition has been suggested as an important environmental factor other than viruses and chemicals in the pathogenesis of type 1 diabetes (T1D). Whereas various maternal dietary nutritional elements have been suggested and examined in T1D of both humans and experimental animals, the results largely remain controversial. In a series of studies using T1D model nonobese diabetic (NOD) mice, maternal dietary n-6/n-3 essential fatty acid ratio during pregnancy and lactation period, that is, early life stages of the offspring, has been shown to affect pathogenesis of insulitis and strongly prevent overt T1D of the offspring, which is consistent with its preventive effects on other allergic diseases.

## 1. Introduction

**Figure 1:**
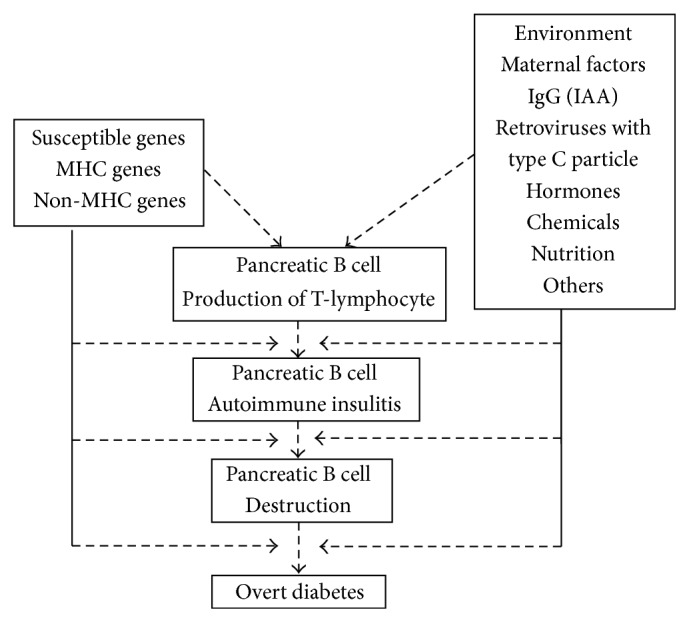
Mechanism on the development of type 1 diabetes (modified from [38, 63]).

Type 1 diabetes (T1D) results from insulin deficiency mostly due to the autoimmune-mediated destruction of the insulin-producing pancreatic islet *β* cells (insulitis) and arises from incompletely understood interactions between *β* cells, the immune system, and the environment in genetically susceptible individuals [1–4] (Figure 1). Concordance of T1D between the monozygotic twin pairs has been reported as approximately 30%, and therefore significant involvement of environmental factors has been implicated in the pathogenesis and clinical course of T1D [5]. Possible mechanisms of environmental factors to destroy islet *β* cells are (1) direct attacking to kill *β* cells, (2) destroying *β* cells to present autoantigens and induce autoimmunity against *β* cells, (3) inducing autoimmunity by molecular homology between the environmental factor and *β* cell antigens, (4) perturbing the immune system to induce autoimmunity against *β* cells, and (5) combinations of the above mechanisms [6]. Environmental factors include viruses, chemicals, and nutrition elements. Fulminant T1D, which constitutes 15–20% of T1D cases with ketosis-onset or ketoacidosis-onset in Japan, is associated with symptoms of common cold such as fever and cough [7], and viral infection has been suggested as the causative factor. A number of viruses have been suggested as environmental factors related to T1D [8]. It has been reported that, in infant T1D patients, Th1 type immune response against coxackie B4 virus, that is, interferon *γ* production and* Tbet* gene expression, is decreased [9]. T-bet is a type-1 transcription factor that regulates the development of type-1 T cell and type-1 antitumor immunity. T-bet expression in dendritic cells is required for the ability of these antigen-presenting cells to prime type-1-polarized T cell responses. Further, RNA of enterovirus has been detected during the acute phase of T1D occurrence in some reports [10]. Whereas many candidate viruses have been detected as infected at the onset of T1D and existence of the virus have been repeatedly proved in the islets, they have not yet been evidenced as the genuine causes of T1D. Among chemicals, cyclosporin [11], an immunosuppressant, and atorvastatin [12], a hydroxymethylglutaryl-CoA reductase inhibitor, are known to be toxic to pancreatic islets and to induce diabetes. Streptozotocin, an alkylating anticancer agent, has been frequently used to cause diabetes in experimental animals [13–15].

A number of nutrients such as cow's milk protein, activated vitamin D, vitamin C, and polyunsaturated fatty acids have also been suggested as environmental factors that affect or are involved in the pathogenesis of T1D which is recently increasing in the incidence. In humans, breast feeding has been reported to reduce the incidence of T1D, while other reports did not find significant difference in the ratio of breast feeding between T1D children and control children [16]. Bovine serum albumin has been suggested as the environmental factor involved in the possible relation between cow's milk intake and T1D occurrence to induce autoimmunity against islet cells based on its molecular homology with pancreatic autoantigens [17]; however, the causative relation still remains controversial [16–19]. Vitamin C is expected to protect islet cells from cytotoxic effects of free radicals [20]. Multiple chemical compounds such as oxidizers, antioxidants, and synthetic coloring agents are involved in foods, and they may be separately or in combination cytotoxic to islet cells or predispose islet cells to be seriously damaged by other factors [20]. In this review, we describe maternal factors, especially nutritional factors, that are suggested to affect development of insulitis and clinical course of T1D.

## 2. Maternal Factors during Early Life Stages in the Pathogenesis of T1D

“Maternal factors” have been suggested as environmental factors in the pathogenesis of T1D before and after birth of the offspring in T1D model nonobese diabetic (NOD) mice [21–23] (Figure 1). The inbred NOD mouse strain originated as a hyperglycaemic substrain of the CTS (cataract-prone mouse) at the Shionogi Laboratories, Fukushima-ku, Japan. At the time of weaning, NOD mice develop insulitis that progresses at approximately 100 days of age to invasive insulitis and complete *β*-cell destruction. Although NOD mice have an increased genetic susceptibility to T1D, the penetrance of disease can be modulated by various environmental factors. Hence not all NOD mice in a colony will develop T1D. A number of T1D-susceptibility genes identified in NOD mice (designated* Idd*) have been found to contribute to T1D susceptibility in humans (designated* IDDM*) [24]. Among “maternal factors” are vertical viral infection from mother to offspring and nutrients via placenta and milk, as well as hormones, and insulin autoantibodies which are transmitted from mothers to offspring [21, 25]. Regarding viral infection, viral particles have been observed in islet cells and suggested to be related with inflammation by autoimmune response, and further involvement of hormones from mother has been suggested in the viral induction and associated progression of insulitis [22, 23, 26, 27]. Insulin autoantibody (IAA) is a potent predictive marker of T1D and has been frequently examined in large scale studies and reported to become positive early after birth both in humans and mice [21, 25]. Greeley et al. [21] reported that IAA can be transmitted from IAA-positive pregnant female T1D model NOD mice to the offspring to cause T1D. However, we and other groups have obtained contradictory results and thus maternal IAA transmission to the offspring and its involvement in the pathogenesis of T1D still remain controversial [21–23, 25].

Regarding effects of nutrition via mother, relation between breast feeding or cow's milk protein intake during lactation period and incidence of T1D has been suggested both in humans and experimental animals [28, 29]. Type 1 Diabetes Prediction and Prevention Project (DIPP) performed HLA typing using the cord blood of newborn babies and examined autoantibodies in the high-risk babies of T1D [29]. They reported that early exposure to formula tended to increase both the ratio of positive autoantibodies and incidence of T1D [29]. Relation between fish oil and T1D has been examined in large scale studies; however, it still remains controversial. While vitamin D, or the n-3 fatty acids, that is, eicosapentaenoic acid (EPA) and docosahexaenoic acid (DHA), has been suggested to be related to IAA level and to decrease incidence of T1D, results appear to vary depending on the period and amount of nutrients [30–35]. In addition to “exogenous” maternal factors such as nutrition in the maternal diet, “endogenous” maternal factors based on the genetic constitution have to be considered in the pathogenesis of T1D in the offspring. We previously showed using mutual transfer of embryos into uterus between NOD and two other genetically different nondiabetic mice that “maternal factors” as a whole strongly affect the onsets and ratios of insulitis and overt diabetes [22]. Since NOD and the other two strain mice were maintained and used in the study under the same conditions including foods and water [22], genome-based maternal factors seem to play important role in the observed difference in the onsets and ratios of insulitis and overt diabetes.

## 3. Nutrition as Environmental Factors in the Early Life Stages

Nutrients in foods are essential to our life through all the life stages. However, required amounts and kinds of nutrients vary depending on each life stage from prenatal to old age periods and may also be different to effectively prevent diseases including T1D depending on the life stages as suggested in reports [36–39]. Several dietary manipulations have been tested as interventions, including infant formulas free of either cow's milk or of bovine insulin, infant formula supplemented with DHA, delayed introduction of gluten-containing foods, and vitamin D supplementation. Secondary prevention studies have been conducted in both children and adults with diabetes autoantibodies. Interventions tested include nicotinamide, insulin injections, oral insulin, nasal insulin, glutamic acid decarboxylase, and cyclosporine [39].

Peppa et al. [36] reported that T1D incidence in NOD mice was evidently increased when advanced glycation end-products (AGEs) were administered during the prenatal and newborn period. Fu et al. [37] showed that epigallocatechin when administered after weaning decreased the occurrence of T1D in NOD mice. We also previously showed that n-6/n-3 ratio of unsaturated fatty acids in the maternal diet during gestation and lactation rather than that of offspring after weaning significantly modified incidence of overt diabetes in NOD mice [38].

## 4. Effects of Dietary Essential Fatty Acid on the Inflammation and Immune Response

Since mammals require but cannot synthesize fatty acids with double bonds distal to the ninth carbon atom, long chain polyunsaturated fatty acids are essential to their diet [40]. Linoleic acid (18 : 2 n-6) is a major essential fatty acid found in oils derived from plant seeds such as corn or safflower. Linoleic acid can be elongated and desaturated to yield arachidonic acid (20 : 4 n-6; AA). The action of Δ15-desaturase in plants converts linoleic acid to *α*-linolenic acid (18 : 3 n-3) which can be elongated to EPA (20 : 5 n-3) and DHA (22 : 6 n-3). These latter conversions to EPA and DHA occur slowly in mammals but are carried out readily by marine algae. Transfer of EPA and DHA from these algae through the food chain to fish makes fish oil the primary source of highly unsaturated n-3 polyunsaturated fatty acids in the human diet as well as dietary supplements [41].

The capacity of n-3 essential fatty acid to modulate immune function and suppress inflammatory responses has been reviewed extensively [42–45]. N-3 essential fatty acids suppress proinflammatory cytokine production, lymphocyte proliferation, cytotoxic T cell activity, natural killer cell activity, macrophage-mediated cytotoxicity, neutrophil/monocyte chemotaxis, MHCII expression, and antigen presentation. Evidence that these cellular effects indeed impact immune function* in vivo* is reflected in n-3 essential fatty acid attenuation of mediator production, leukocyte homing, delayed-type hypersensitivity, allograft rejection, and acute inflammatory responses in experimental animals in which human inflammation and autoimmune diseases are modeled. N-3 essential fatty acids appear to mediate these pleiotropic effects via both eicosanoid-dependent and eicosanoid-independent pathways.

Animal studies have demonstrated the potential for n-3 essential fatty acid to suppress onset (e.g., aberrant Ig production and glomerular deposition) and progression (e.g., inflammation, glomerular injury, and proteinuria) of disease in several animal models of autoimmune glomerulonephritis. However, major gaps still exist in our understanding of the precise molecular mechanisms of the effects of n-3 fatty acids as well as identities of their cellular targets [46].

Maternal intake of fatty acids during pregnancy was associated with childhood asthma. Maternal *α*-linolenic acid, total n-3 essential fatty acid, and palmitic acid intake may decrease, while arachidonic acid intake may increase the risk of asthma in the offspring [47].

An observational study suggested that dietary intake of n-3 fatty acids is associated with reduced risk of islet autoimmunity in children at increased genetic risk for type 1 diabetes [28]. The Trial Net Nutritional Intervention to Prevent (NIP) type 1 Diabetes Pilot Trial is assessing the feasibility of implementing a full-scale study to determine if nutritional supplements with DHA during the last trimester of pregnancy and the first few years of life will prevent the development of islet cell autoimmunity in children at high risk for type 1 diabetes [48].

The data from the Finnish type 1 Diabetes Prediction and Prevention Nutrition Study suggest that maternal consumption of butter, the ratio of n-6 : n-3 fatty acid, and intake of polyunsaturated fatty acid and *α*-linolenic acid during pregnancy may be potential determinants of allergic rhinitis in the offspring [49].

In the next section, we review the relation between maternal factors, especially maternal and offspring dietary n-6/n-3 ratio of unsaturated fatty acids, and pathogenesis and clinical course of T1D in NOD mouse offspring.

## 5. Effects of Maternal Dietary Essential Fatty Acid Ratio on the Pathogenesis and Clinical Course of T1D of Offspring

Nutrients including essential fatty acids are not only transmitted to offspring via mother but also may affect maternal immune and endocrine systems and thus may affect cytokines, antibodies, and hormones that are transmitted to the offspring [39–41]. n-6/n-3 essential fatty acid ratio in diet has been reported to increase in Europe and USA, and the average in USA is reported from 12 to 15 [50]. While n-6/n-3 ratio remains from 2 to 3 among Japanese who frequently eat fish, it is increasing to 8 or 9 among young Japanese whose diet is being westernized [50]. The increase in n-6/n-3 ratio has been suspected as being involved in the recent increase in allergic, vascular, and immunologic diseases [51]. Experimental animal studies showed that fat intake even before pregnancy affects its involvement in the fetal tissue and that n-6/n-3 ratio plays important roles in histogenesis, growth, and development of immune system during fetal and neonatal periods [52, 53].

**Figure 2:**
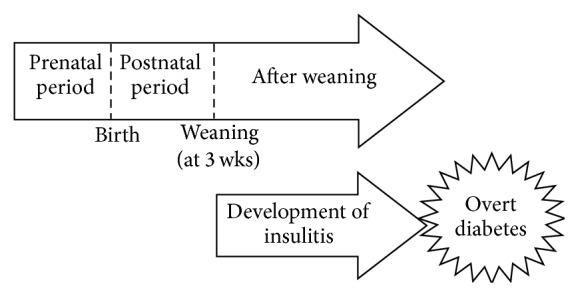
Life stages of NOD mice Type 1 diabetes results from insulin deficiency, mostly due to the autoimmune-mediated destruction of the insulin-producing pancreatic islet *β* cells (insulitis) (modified from [64]).

NOD female mice mostly start to show insulitis after weaning, and 100% of individuals have insulitis by 12 weeks of age (Figure 2). Among chemicals and nutrients studied using NOD are cyclosporin and streptozotocin whose effects on the development of insulitis were examined after 8 weeks of age, whereas barley protein's effects on the induction and suppression of insulitis differ depending on the timing of exposure [54]. We have performed series of studies to investigate the effect of maternal or postweaning offspring's nutrition in particular n-6/n-3 ratio on the development of insulitis and T1D [38, 55, 56]. In these studies, NOD mothers were fed with the randomly assigned chow for the gestation period for longer than 4 weeks before being mated and becoming pregnant. We prepared two kinds of chows with different n-6/n-3 ratios, “low-n-3” chow (L: n-6/n-3 = 14.5) or “n-3” chow (n: n-6/n-3 = 3.0), and provided them to NOD female mice (1) four weeks before pregnancy through gestation, (2) during lactation after birth of their offspring, and (3) to the offspring after weaning until the end of the experiment [38, 55, 56]. We showed that different n-6/n-3 ratios modified the incidences of insulitis and overt diabetes and suggested that DHA and EPA have to be included in the diet to cause the effect [38, 55, 56].

**Figure 3:**
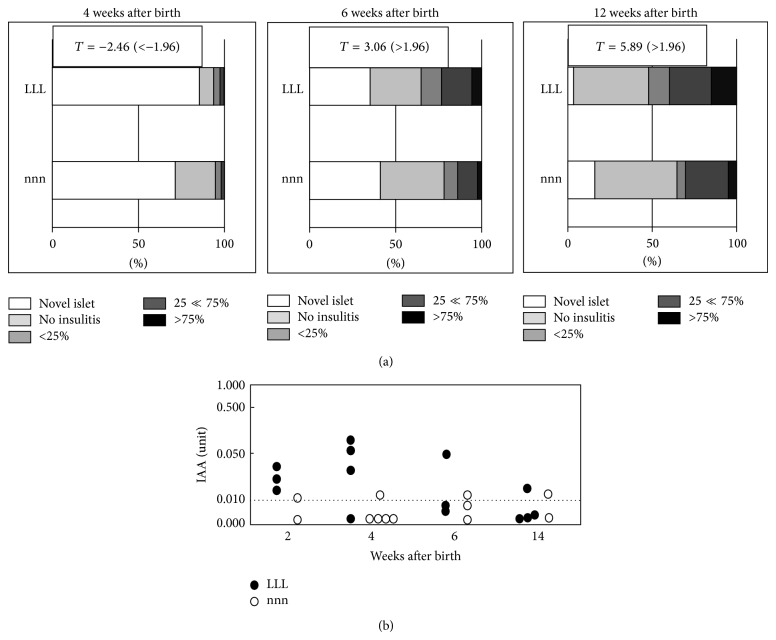
Levels of insulitis (a) and serum insulin autoantibody (IAA) levels (b) in the offspring at different ages (modified from [38]).

**Figure 4:**
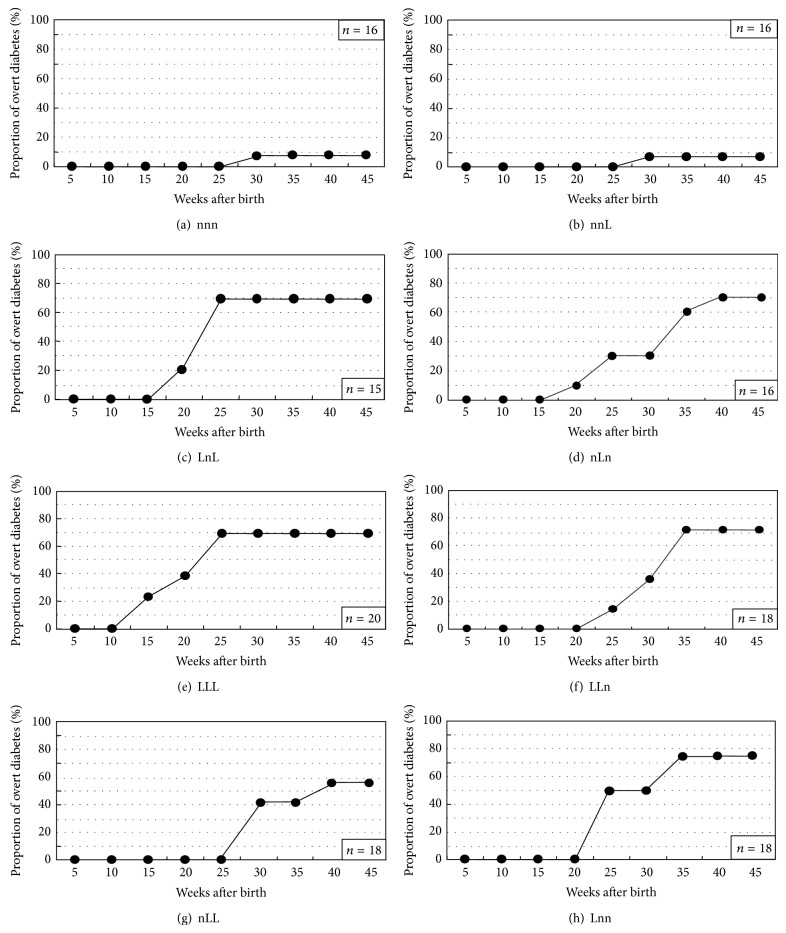
Effects of combination of n-3(n) and low n-3(L) chows on the onset and incidence of overt diabetes in NOD mice [38, 63].

We further used L chow that induced the highest incidence of overt diabetes and n chow that prevented overt diabetes to the lowest incidence to examine in detail the effects of different n-6/n-3 ratios in nutrition on T1D depending on the different life stages (prenatal, lactation, and postweaning periods) [38]. In the n-chow-fed offspring from n-chow-fed mother (nnn), levels of insulitis were higher than those in the L-chow-fed offspring from L-chow-fed mother (LLL) at 4 weeks of age, while the levels in the nnn offspring became lower than those in the LLL after 6 weeks (Figure 3(a)). Early IAA expressions were observed from 2 to 6 weeks in the LLL offspring but not in the nnn (Figure 3(b)). The nnn offspring (Figure 4(a)) exhibited strong suppression of overt diabetes development in regard to the onset and accumulated incidence of diabetes compared to the LLL (Figure 4(e)). The study with different combination of n and L chows during gestation and lactation in mother and in postweaning offspring revealed that the only nnL-chow in which n chow was provided through prenatal to lactation periods (Figure 4(b)) significantly suppressed the development of diabetes with similar kinetics to nnn-chow, whereas the other combinations may delay the onset of diabetes (Figures 4(f), 4(c), 4(d), 4(g), and 4(h)). These results indicated that n-6/n-3 ratio in the maternal diet during pregnancy and lactation period affects the development of insulitis and overall incidence of overt diabetes [38] and thus strongly suggest that diets with appropriate n-6/n-3 ratios should be provided during the appropriate life stages to prevent T1D.

T helper type 17(Th17) cells have been shown to play important roles in mouse models of several autoimmune diseases that had previously been thought to be Th1-dominant. In the NOD mouse, however, the relevance of IL-17/Th17 is still controversial, because of their inherent plasticity. Th17 cells derived from BDC2.5 NOD mice transfer diabetes through conversion to Th1 cells* in vivo* [57]. Allen et al. reported that n-3 essential fatty acids reduce CD4(+) T-cell activation and differentiation into pathogenic Th17 cells by 25–30% [58]. Their data suggested that n-3 essential fatty acids suppress Th17 cell differentiation in part by reducing membrane raft-dependent responsiveness to IL-6, an essential polarizing cytokine [58]. Kuriya et al. studied the impact of IL-17 single-deficiency or IL-17/IFN-*γ* receptor double-deficiency on the development of insulitis/diabetes in NOD mice [57]. IL-17 single-deficiency significantly delayed the onset of diabetes and attenuated the severity of insulitis, but the cumulative incidence of diabetes until 50 weeks of age in IL-17 deficient mice was similar to that in wild-type (wt) mice. Adoptive transfer study with CD4+CD25−T cells from young nondiabetic IL-17 single-deficient NOD mice, but not that from older mice, showed also significantly delayed disease in the recipient NOD-Scid mice as compared with those from the corresponding wt mice. On the other hand, IL-17/IFN-*γ* receptor double-deficiency significantly suppressed the development of diabetes, although the levels of insulitis were similar between single-deficient and double-deficient mice. Kuriya et al. reported that IL-17/Th17 plays a significant role in the development of insulitis in prediabetic NOD mice and the interaction of Th1-Th17 cells contributes to diabetes development [57].

Taken together with these reports, data from our NOD mouse studies suggest that low n-6/n-3 ratio diet (a higher ratio of n-3 essential fatty acid) fed during prenatal period and postnatal period before weaning affected Th17 cell differentiation and interaction between Th1 and Th17 cells and eventually prevented development of overt diabetes. Delayed onset of overt diabetes in the groups fed with low n-6/n-3 ratio diet after weaning may also be related with a similar mechanism. We have no clear explanation at present for the earlier onset of insulitis albeit the eventual prevention of overt TID in the nnn group. Norris et al. showed based on the Diabetes Autoimmunity Study in the Young (DAISY) cohort that low erythrocyte membrane docosapentaenoic acid (DPA) levels are associates with an increased risk of developing IAA [59]. Fatty acid desaturase activity varies depending on the life stages and may complicate the relation between n-3 essential fatty acids and development of T1D. Data from our studies also suggest that appropriate dietary n-6/n-3 ratio may differ at each life stage. During the early life stages, that is, prenatal and early postnatal periods, fatty acid desaturase activity is low, and therefore dietary requirement of n-3 essential fatty acids such as DHA and EPA may be higher to prevent development of autoimmune responses.

## 6. Maternal Prepregnant Environment as Another Life Stage for Offspring

Maternal diet and nutrition are expected to have different influences on the offspring depending on the different life stages for the offspring. Amount and quality of maternal nutrition before pregnancy should have a close relation with maternal endocrine and immune conditions, albeit different from that after pregnancy. Our preliminary study showed that changes in n-6/n-3 ratio in the maternal diet before and after pregnancy affected the incidence and onset time of T1D [60]. As Developmental Origins of Health and Disease (DOHaD) hypothesis provoked by Barker and Osmond [61] and Silveira et al. [62] has been extensively examined, the effects of human maternal environment for the development of disease in different life stages are increasingly reported. Maternal environment including neural, endocrine, and immune systems during pregnancy is based on that before the pregnancy, maternal environment even before the pregnancy has to be considered as another important life stage, in addition to maternal environment by direct relation between mother and offspring via placenta and milk, for the health and diseases of the offspring.

## 7. Conclusion

Among maternal factors, dietary n-6/n-3 EFA ratio during pregnancy and lactation period may affect pathogenesis of insulitis and clinical course of T1D in the offspring.
